# On the mechanisms underlying attenuated redox responses to exercise in older individuals: A hypothesis

**DOI:** 10.1016/j.freeradbiomed.2020.10.026

**Published:** 2020-12

**Authors:** Malcolm J. Jackson

**Affiliations:** MRC-Versus Arthritis Centre for Integrated Research Into Musculoskeletal Ageing (CIMA), Department of Musculoskeletal and Ageing Biology, Institute of Life Course and Medical Sciences, University of Liverpool, Liverpool, L7 8TX, UK

**Keywords:** Aging, Exercise, Training, Muscle, Redox

## Abstract

Responding appropriately to exercise is essential to maintenance of skeletal muscle mass and function at all ages and particularly during aging. Here, a hypothesis is presented that a key component of the inability of skeletal muscle to respond effectively to exercise in aging is a denervation-induced failure of muscle redox signalling. This novel hypothesis proposes that an initial increase in oxidation in muscle mitochondria leads to a paradoxical increase in the reductive state of specific cysteines of signalling proteins in the muscle cytosol that suppresses their ability to respond to normal oxidising redox signals during exercise. The following are presented for consideration:Transient loss of integrity of peripheral motor neurons occurs repeatedly throughout life and is normally rapidly repaired by reinnervation, but this repair process becomes less efficient with aging. Each transient loss of neuromuscular integrity leads to a rapid, large increase in mitochondrial peroxide production in the denervated muscle fibers and in neighbouring muscle fibers. This peroxide may initially act to stimulate axonal sprouting and regeneration, but also stimulates retrograde mitonuclear communication to increase expression of a range of cytoprotective proteins in an attempt to protect the fiber and neighbouring tissues against oxidative damage. The increased peroxide within mitochondria does not lead to an increased cytosolic peroxide, but the increases in adaptive cytoprotective proteins include some located to the muscle cytosol which modify the local cytosol redox environment to induce a more reductive state in key cysteines of specific signalling proteins. Key adaptations of skeletal muscle to exercise involve transient peroxiredoxin oxidation as effectors of redox signalling in the cytosol. This requires sensitive oxidation of key cysteine residues. In aging, the chronic change to a more reductive cytosolic environment prevents the transient oxidation of peroxiredoxin 2 and hence prevents essential adaptations to exercise, thus contributing to loss of muscle mass and function.

Experimental approaches suitable for testing the hypothesis are also outlined.

## Highlights

• It is hypothesised that denervation leads to failed redox signalling and attenuated muscle exercise responses in aging.
• Loss of peripheral motor neurons leads to increased mitochondrial peroxide in denervated and neighbouring muscle fibers.
• This peroxide stimulates increased expression of cytoprotective proteins to protect the fiber against oxidative damage.
• Increased mitochondrial peroxide does not increase cytosolic peroxide, but adaptations reduce the cytosol redox state.
• Key adaptations of skeletal muscle to exercise involve transient peroxiredoxin oxidation in the cytosol.
• Peroxiredoxin oxidation is suppressed during aging by a chronic change to a more reductive local cytosolic environment.

## Graphical abstract

**Figure:**
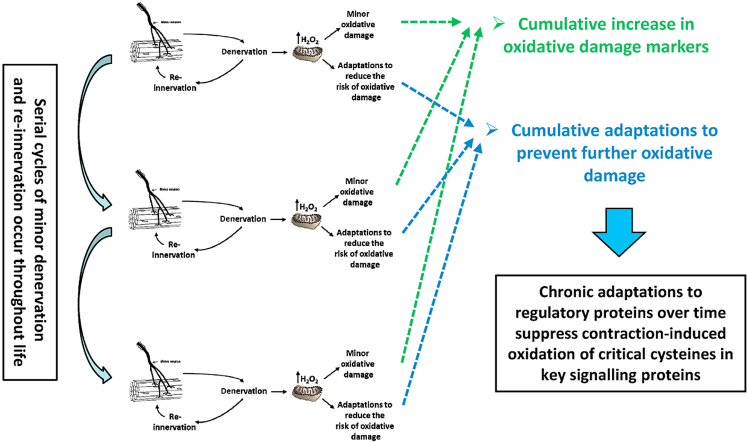

The social and medical issues associated with increasing numbers of elderly members of populations are affecting most countries. The chronic conditions associated with musculoskeletal aging contribute to a heavy functional and economic burden for rapidly aging populations. Age is the greatest risk factor for the common musculoskeletal disorders, osteoarthritis, osteoporosis and sarcopenia and the number of older people with such disorders in countries such as the UK continues to increase dramatically [1]. There remains a clear need to identify and test new strategies to reduce the incidence, and consequences, of common age-related chronic disease. This is particularly acute for debilitating age-related disorders of the musculoskeletal system since these have major adverse effects on independence and quality of life of older individuals and which, by limiting physical activity, amplify age-related risks of multiple cardio-metabolic diseases, major cancers and neurodegenerative diseases [2,3]. This paper will briefly review published data and our own studies on the role that an age-related failure of beneficial adaptations to exercise play in age-related loss of skeletal muscle mass and function (sarcopenia), present a novel hypothesis of the mechanisms by which this occurs and outline experimental approaches suitable for testing the hypothesis.

## 1 Aging is associated with loss of muscle fibers, weakness of the remaining fibers and loss of motor units

In older people, declining muscle mass and function causes instability and increased risk of falls with a loss of independence [4]. By age 70, the cross-sectional area of skeletal muscle is reduced by 25–30% and muscle strength by 30–40% [5]. The reduction in muscle mass and function with age in humans and rodents is due to a decrease in the number of muscle fibers, and atrophy and weakening of those remaining [[6], [7], [8]]. Intrinsic and extrinsic changes regulating muscle aging in humans also occur in rodents, indicating that mice and rats are relevant models of human sarcopenia [9,10].

The loss of muscle that occurs with aging occurs in parallel with loss of motor units in both humans and rodents [11,12]. In young and adult humans and animals, damage to terminal axons and motor unit turnover occurs during everyday activities and loss of innervation is repaired by sprouting and regrowth of axons from the damaged nerve leading to rapid re-innervation of neuromuscular junctions (NMJ) [13]. With increasing age, re-innervation does not occur appropriately and re-innervation by sprouting from adjacent axons or NMJ can occur [14]. The effect of this is increased grouping of fibres of a single fibre type [15] with the formation of “giant” motor units which are eventually lost [16]. A 25–50% reduction in the number of motor neurons occurs in both man and rodents with aging [17,18] and there is evidence for selective loss of large fast α-motor neurons occurs leading to an apparent increased proportion of type I (slow twitch) muscle fibres, that is particularly apparent in humans [19,20]. Various researchers have reported loss of innervation of individual fibers in aged muscles, including our study which indicated that ~15% of individual muscle fibers from old mice are completely denervated and ~80% of NMJs showed some disruption [21]. Maintenance of the NMJ is increasingly seen to be key to interventions that may maintain muscle mass and function in older age [22].

## 2 Exercise plays a significant role in maintaining muscle mass at all ages

Skeletal muscle adapts to different forms of exercise in many positive ways including an increase in aerobic capacity, increased muscle force generation, increased mass and decreased fatigability. These processes are essential for maintenance of muscle mass and function at all ages, but this becomes increasingly important with increasing age [23,24]. The mechanisms underlying the beneficial adaptations to exercise have been the subject of a number of studies and key pathways have been identified that provide potential targets for interventions aimed at optimising the beneficial effects of exercise [25].

Despite these substantial advances there is still a lack of full understanding of the specific changes that occur in muscle during exercise to trigger the signalling pathways leading to these adaptations. Reactive oxygen species (ROS), and specifically hydrogen peroxide (H_2_O_2_), have been proposed as key factors that stimulate adaptive changes in contracting skeletal muscle [[26], [27], [28]].

## 3 Inhibitor studies indicate that the range of adaptations to exercise stimulated by ROS is extensive

Muscle fibers respond to contractile activity by an increase in the intracellular generation of superoxide and nitric oxide (NO) with the formation of secondary ROS and reactive nitrogen species [26,29,30]. The importance of these as physiological signalling molecules with regulatory functions that modulate changes in cell and tissue homeostasis and gene expression has become increasingly apparent [[31], [32], [33]]. Signalling by these reactive molecules is mainly achieved by targeted redox modifications of specific residues in proteins [34,35].

Many studies have examined the effects of high doses of single antioxidant nutrients, or mixtures of these in rodents and humans undertaking various exercise protocols. The data obtained have been variable, but many of these studies demonstrated that antioxidants inhibited cytoprotective responses, such as the increase in heat shock and other stress proteins [36,37] that followed exercise, inhibited mitochondrial biogenesis [[38], [39], [40]], prevented the beneficial increase in muscle insulin sensitivity [38] and inhibited the release of cytokines and inflammatory mediators [41]. While these studies have been subject to controversy [42,43], these data support the possibility that ROS act as beneficial signalling molecules that mediate multiple adaptations to exercise.

## 4 Key signalling pathways involved in muscle adaptations appear to be redox regulated

**Fig. 1:**
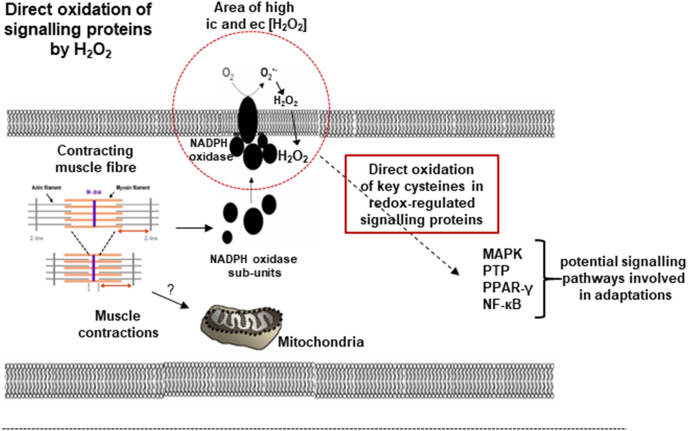
Potential mechanisms underlying generation of ROS (superoxide and H_2_O_2_) during muscle contractions and activation of key redox-sensitive signalling molecules involved in muscle adaptations to the contractile activity. From Ref. [44].

Studies have identified several key signalling pathways involved in skeletal muscle responses to contractile activity that appear to be redox regulated, although the exact mechanisms and proteins involved remain unclear. Examples of signalling pathways that are activated in muscle by contractile activity and inhibited by antioxidants include Mitogen-activated protein kinases (MAPK), Protein tyrosine phosphatases (PTP), Peroxisome proliferator-activated receptor gamma (PPAR-γ) and Nuclear factor-κB (NF-κB) [44] (Fig. 1). Initial comparisons of studies showing activation of signalling pathways by H_2_O_2_ in cell culture and activation of these same pathways by contractile activity in muscle *in vivo* also suggested a potential role for H_2_O_2_ in activation of these pathways during exercise. However, detailed examination of the likely *in vivo* concentrations of H_2_O_2_ with those used to activate key signalling molecules in cell culture have cast doubt on this possibility [44]. In brief, H_2_O_2_ has been shown to activate NF-κB [45,46], p38-MAPK [47] and many other signalling molecules [48], but key cysteines in the signalling molecules examined are relatively unreactive with H_2_O_2_ and typical concentrations of H_2_O_2_ used to activate them are in the range 10^−4^-10^−3^ M. In contrast intracellular H_2_O_2_ concentrations are in the order of 10^−9^-10^−8^ M [49] and during contractions, muscle intracellular H_2_O_2_ concentrations may increase by a maximum of 10^−7^ M [44,50].

## 5 Aging induces a failure of the normal physiological roles of ROS in redox signalling of adaptations to contractile activity

Studies in many cell models and tissues have indicated that ageing is associated with a failure of adaptations to stress [51]. In skeletal muscle this is seen as an attenuation of important adaptations to exercise including acute stress responses [52], mitochondrial biogenesis [10,53,54] and anabolic responses [55]. Such changes reduce the efficacy of exercise in maintaining muscle and our previous data indicate that transgenic approaches to correct specific responses that are attenuated during ageing can help maintain muscle mass and function [[56], [57], [58]].

Several of these ROS-stimulated responses are attenuated in old mice including increased stress responses [52] and mitochondrial biogenesis [54,59]. Studies in humans indicate that the age-related attenuation in redox-mediated responses to exercise also occurs, although a small number of such responses may be maintained by long term exercise training [10].

## 6 Mechanisms underlying attenuation of redox signalling in skeletal muscle with age

A number of pieces of evidence suggest that a chronic increase in H_2_O_2_ generation by muscle mitochondria leading to increased oxidation causes the attenuation of redox-mediated adaptations to contractile activity that is seen with aging. Studies using the non-specific ROS probe dichloro-dihydro-fluorescein (DCFH), indicated a chronic increase in ROS activities occurred in muscle from old mice at rest with no further increase following contractions [60] and we speculated that the chronic increased oxidation was derived from increased mitochondrial ROS generation, We further speculated that this increased oxidation leads initially to chronic activation of transcription factors which ultimately become insensitive to further changes in ROS [50]. Attenuated adaptive responses are also seen in mice lacking Cu, Zn superoxide dismutase (SOD1null mice), a model of accelerated muscle aging where muscle mitochondrial ROS generation is also elevated [61]. In contrast, Martinez-Guimera and colleagues developed a molecular model to explain how chronic increased ROS activities might interfere with redox signalling pathways [62]. They identified that, in a process termed “molecular habituation”, a sustained ROS signal reduced the responsiveness of signalling pathways through prolonged activation of negative regulators, such as occurs in aging with upregulation of regulatory proteins for ROS, including catalase, glutathione peroxidase1, thioredoxin (Trx)1 and 2, and peroxiredoxins (Prx) 3–6 [60,63,64].

## 7 Previous studies have attempted to ameliorate aging pathologies by decreasing oxidative damage

Tissues of aged organisms contain greater oxidative damage to lipids, DNA and proteins compared with young organisms [[65], [66], [67]]. Although initial interventions to reduce ROS activities in non-mammalian models were reported to extend lifespan [[68], [69], [70], [71]] these effects were not confirmed [72] and it is clear that the level of ROS generation is not a fundamental determinant of lifespan. Despite this, some authors have argued that the age-related changes in ROS activities and oxidative damage are important mediators of age-related disorders [73]. Mitochondrial peroxide generation has been repeatedly reported to be increased in skeletal muscle during aging [65,74].

## 8 How does the increase in mitochondrial ROS generation occur in aging muscle?

A great deal of research has focussed on the potential causes and mechanisms of the increased mitochondrial ROS generation in tissues during aging (e.g. see reviews [67,75,76]). There is some controversy in the literature concerning the main sites for the reported increases in mitochondrial ROS generation during aging, but most studies indicate that excessive generation of superoxide from electron chain complexes is the likely source with complexes I, II and III having the greatest capacity for generation [75].

## 9 Role of denervated fibers in increased mitochondrial ROS generation in skeletal muscle of old mice

**Fig. 2:**
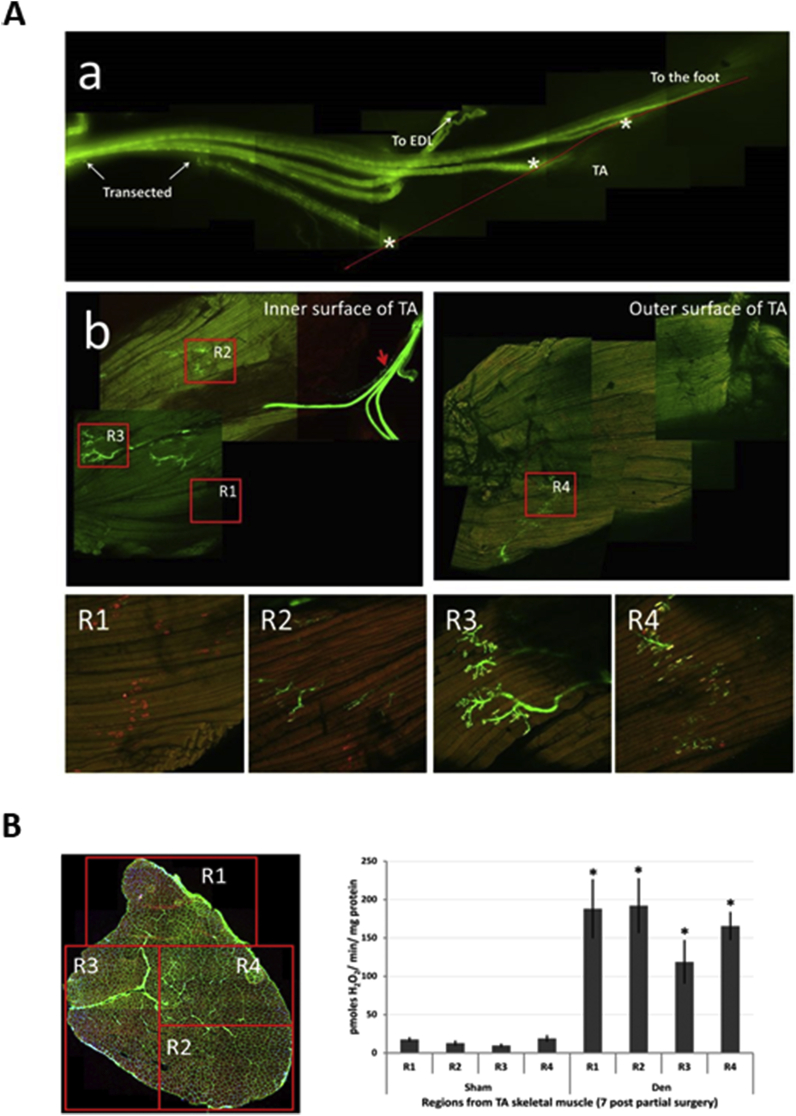
**A**. Images of the peroneal nerve in Thy1-YFP mice to show one of the 3 branches of the peroneal nerve (indicated by * in the figure) transected immediately prior to entry into the Tibialis Anterior (TA) muscle leaving 2 branches intact. This image was taken at 7 days post-transection showing lack of any regrowth of the nerve (a). Intact muscles stained with α-bungarotoxin to visualise the AChR showed variability in NMJ structure (b). At 7 days post-surgery, four regions were identified where: all fibers had lost axonal input but retained AChR (R1), all fibers retained full innervation (R3), or fibers had a mix of innervated and some denervated fibers (R2, R4). Reproduced from Ref. [79]. **B**.The regions identified in Fig. 2A in longitudinal section were identified on transverse sections of the TA muscle and small bundles of fibers were obtained from each region (b). These fibers were permeablised and state 1 mitochondrial peroxide generation examined at 7 (b) days post-surgery in comparison with sham-operated control muscles; *P < 0.05 compared with fibers from the same region of sham-operated muscles. Reproduced from Ref. [79].

The role of motor neurons and NMJ in regulation of muscle ROS generation and modified redox status in old mice has only recently become recognised. Muscle of older humans and rodents show substantial loss of NMJ integrity [21,77]. Transection of the innervating nerve was found to cause a large increase in peroxide generation by mitochondria of the denervated muscle [78]. The original authors of this work implicated the increased mitochondrial peroxide in stimulation of degenerative pathways in the denervated muscle. We have examined the effect of partial denervation of the mouse tibialis anterior (TA) muscle and showed a substantial increase in mitochondrial peroxide generation in the denervated fibers and also in neighbouring innervated fibers in the same muscle [79] (Fig. 2). These data suggest that the loss of innervation seen in some muscle fibers during aging may contribute to increased mitochondrial ROS generation previously reported in muscle from old animals and humans [79]. Increased peroxide generation by muscle mitochondria is also seen in mice with whole body deletion of SOD1 which show an accelerated muscle aging phenotype and premature age-related loss on motor neurons and disruption of NMJ [80]. This is associated with increased muscle oxidative damage and attenuated muscle responses to contractile activity [61]. In contrast, mice with muscle specific deletion of SOD1 showed no premature loss of muscle mass, normal mitochondrial peroxide generation and an unaltered response to contractile activity in comparison with wild type mice [81] but transgenic restoration of SOD1 only in motor neurons of whole body SOD1 null mice (a “nerve rescue” model) completely eliminated the neuromuscular phenotype seen in the whole body knockout and restored muscle mitochondrial peroxide generation to normal levels [82]. Taken together these studies indicate that motor neuron integrity can play a role in regulation of mitochondrial ROS generation in skeletal muscle [83,84].

Some authors have proposed that the denervation-induced increase in mitochondrial peroxide generation stimulated degenerative pathways in the muscle [78,85,86]. There is also evidence that the increased peroxide may initially reflect a muscle attempt to stimulate re-innervation through promotion of axonal sprouting and nerve regrowth [87,88], may mediate remodelling of NMJ [89], and stimulate adaptations that modify the muscle content of multiple proteins to prevent oxidative damage to the muscle fiber itself [90]. Data from this latter paper also indicate that proteins showing modified expression following increased mitochondrial peroxide generation are localised in both mitochondria and the cytosol providing some evidence for redox cross-talk between oxidised mitochondria and cytosol. The extent to which a large increase in mitochondrial ROS generation can influence cytosolic redox status has not been extensively studied, although it appears to have generally been assumed that an increase in mitochondrial ROS generation will lead to an increase in oxidation in the cytosol [91].

## 10 Intracellular compartmentalisation of ROS in muscle fibres

**Fig. 3:**
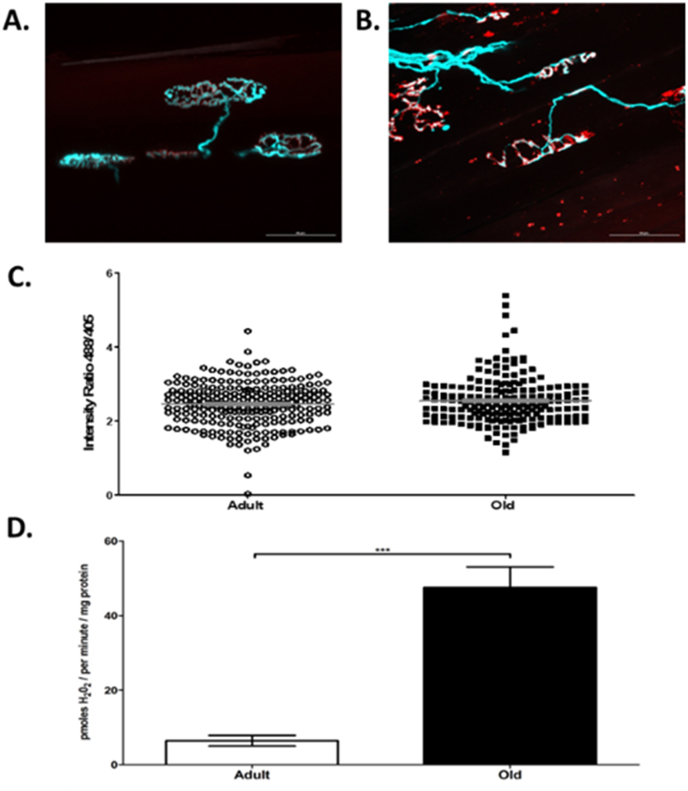
**A**.Representative fluorescent images of NMJ's from adult (6–8 months) (A) and old (26 months) (B) Thy1-CFP mice showing the pre-synaptic terminal motor nerves (blue) and motor endplates stained with α-bungarotoxin (red) (scale bar = 50 μm). No significant difference was observed between the cytosolic H_2_O_2_ content (indicated by the *cyto-HyPer2* fluorescence) of transfected AT muscles from adult and old mice when assessing the ratio of emissions at 516 nm after 488 and 405 excitation (488/405 ratio) from individual fibers (C). Mitochondrial peroxide generation monitored by the rate of amplex red oxidation (expressed as H_2_O_2_ generation) during state 1 respiration (i.e. in the absence of added substrates) from permeablised AT fibers from adult and old mice (D). ***p < 0.001 Reproduced from Ref. [94]. (For interpretation of the references to colour in this figure legend, the reader is referred to the Web version of this article.)

Studies using the ROS-sensitive fluorescent probes (mitoSox and dihydroethidium) localised to different intracellular sites in muscle concluded that the major site for generation of superoxide and hydrogen peroxide by muscle during short term contractions was a plasma membrane or T-tubule localised NADPH oxidase [92,93] with little contribution or effect on mitochondrial ROS. Furthermore, where mitochondrial ROS generation was specifically elevated using antimycin A, no effects on cytosolic ROS activities were seen [92]. Our recent studies have directly compared muscle mitochondrial peroxide generation (measured *ex vivo* by amplex red oxidation in permeablised fibers of the TA muscle) with cytosolic H_2_O_2_ content measured in individual TA muscle fibers *in vivo* using *intra vital* confocal microscopy following transduction of fibers with *cyto-HyPer2* (a specific probe for H_2_O_2_). Data from muscles of old mice show a discrepancy between mitochondrial peroxide generation (which was substantially increased in muscle of old compared with adult mice) and cytosolic H_2_O_2_ content where no increase was seen in individual muscle fibres from old compared with adult mice (Fig. 3) [94]. Thus, these data show clear compartmentalisation of ROS activities with preservation of the muscle cytosolic H_2_O_2_ content in the presence of substantial increases in mitochondrial peroxide generation in aging.

The conclusion that there is sophisticated differential control of the regulation of ROS between cellular compartments to regulate redox signalling and protect against oxidative damage is supported by experiments that used other approaches to oxidise mitochondria [95,96]. These studies also showed that mitochondrial oxidation induced increased nuclear transcription of multiple genes including key enzymes in glutathione synthesis (GCL, GS) leading to increased glutathione in cytosol and mitochondria [96]. These data are compatible with mitochondria signalling directly to nuclei (termed “retrograde mitonuclear communication”) to stimulate nuclear transcriptional programs and to alleviate mitochondrial dysfunction [97]. ROS have been proposed as potential signalling molecules in this process although other pathways, such as calcium, the mitochondrial unfolded protein response (*UPR*^*mt*^), mitochondrial metabolites and mitokines have also been proposed (see review by Merry and Ristow [98]).

Recent studies with the state-of-the-art hydrogen peroxide probe *HyPer 7* targeted to different cell compartments in K562 cells also found that increased mitochondrial H_2_O_2_ generation induced by addition of complex I inhibitors had no effect on the cytosolic H_2_O_2_ content [99]. Furthermore, they concluded from inhibitor studies that the thioredoxin (Trx) system was responsible for the prevention of H_2_O_2_ diffusion from the mitochondrial matrix.

## 11 What is the redox status of the muscle cytoplasm in aging?

**Fig. 4:**
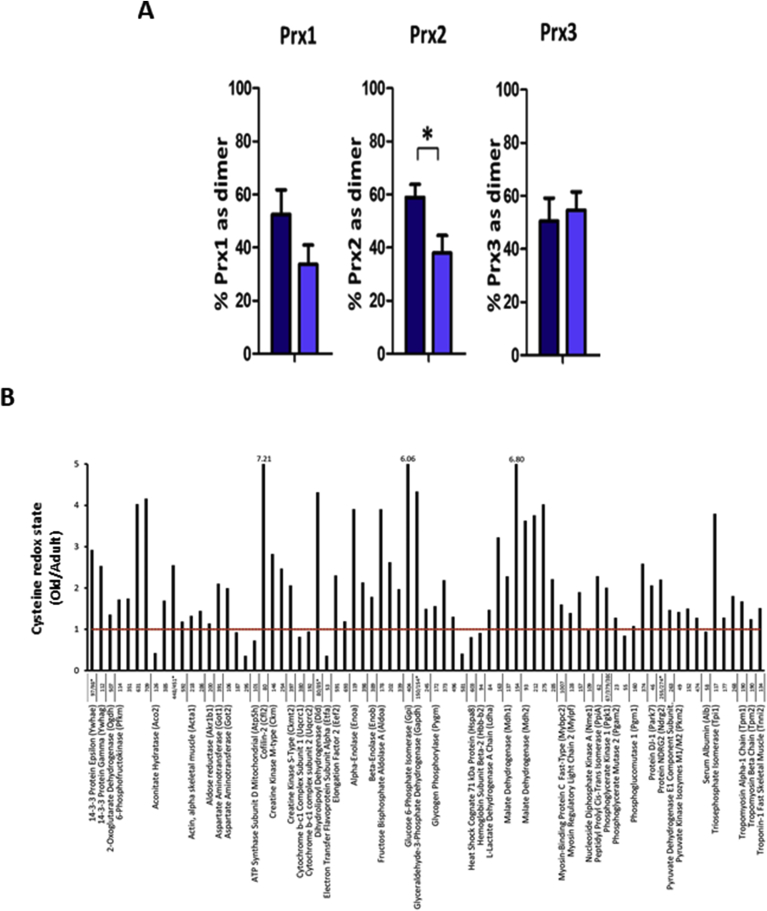
**A**. Percentage of Prx1, 2 and 3 found in the oxidised (dimerised) form on non-denaturing gels from FDB muscle fibers from adult (dark blue bars) and old (light blue bars) mice. The graphs show mean ± SEM. *P < 0.05. Redrawn from Stretton et al., [102]. **B**.Change in the Cys Redox ratio in specific cysteines of peptides found in the gastrocnemius muscles from old and adult mice. Data are expressed as the relative change with increasing age in the reduced/oxidised form (i.e. a ratio greater than one indicates an increased proportion in the reduced form in old mice). Redrawn from Ref. [103]. . (For interpretation of the references to colour in this figure legend, the reader is referred to the Web version of this article.)

Measurements of the oxidation state of different muscle compartments are relatively complex, but can be undertaken by examination of the redox status of proteins localised to the specific organelle or compartment. These include Trx1 (cytosolic and nuclear) compared with Trx 2 (mitochondrial) [63], or peroxiredoxins (Prx) 1 and 2 (cytosolic) compared with Prx 3 (mitochondrial) [100]. Prxs act to scavenge H_2_O_2_ at the low concentrations found in muscle fibers and have been proposed as potential transducers of redox signalling [35,101]. We recently examined Prx1, 2 and 3 oxidation in skeletal muscle myotubes following exogenous additions of H_2_O_2_ or contractile activity. Prx1 and 2 were rapidly oxidised with the formation of dimers following exposure to low external concentrations of H_2_O_2_ (2.5 or 25 μM). Prx3 (localised to mitochondria) was oxidised by 25 μM H_2_O_2_. Exposure to higher concentrations of H_2_O_2_ induced formation of hyperoxidised protein that do not form dimers [102]. Thus, comparison of the proportion of the Prx1, 2 and 3 found in the dimeric or hyperoxidised forms provides a measure of the redox status of different compartments. We have examined this in muscle from adult and old mice. Muscles from old mice showed no increase in hyperoxidised Prx in comparison with adult mice and the level of Prx3 dimerisation was unchanged between adult and old mice, but a greater portion of Prx2 in quiescent muscles of old mice was found in the reduced form (Fig. 4A). Thus, paradoxically, Prx2 in the cytosol of fibers from old mice show less oxidation (i.e. increased reduction) of cysteine thiols compared with adult mice given that ROS levels have been reported to be elevated in muscles from old mice [52,66,67,74].

As an alternative approach to evaluate the redox status of the cytosol compared with the mitochondria, published redox proteomics data have been re-analysed. These data report the proportion of specific protein cysteines in the oxidised or reduced form in muscle from adult or old mice [64,103]. The redox proteomic approach used in these studies involved differential labelling of reduced and reversibly oxidised cysteine residues. The redox state of individual cysteine residues was calculated from the parent ion intensities of cysteine containing peptides labelled with either a light (reduced cysteine residues labelled with *d* (0) *N*-ethylmaleimide (NEM)) or heavy (reversibly oxidised labelled cysteine residues labelled with *d* (5) NEM) alkylating agent. This approach identifies reversibly oxidised cysteine residues, but does not distinguish between potential different reversible cysteine oxidation pathways, such as formation of disulphides, or glutathionylation [64]. Data from Smith et al. [103] obtained from analysis of *gastrocnemius* muscles from adult and old mice are represented in Fig. 4B and illustrate the variability between age-related changes in the level of oxidation of specific cysteine residues. Such variability is not surprising since each cysteine has unique kinetics for oxidation/reduction and differences in cellular location and local environment which influence the level of oxidation [104]. Of the 75 peptides with reversibly oxidised cysteines that were detected in the gastrocnemius muscle, 13 show increased oxidation, while 62 show increased reduction in muscle from old compared with adult mice. These cysteine-containing peptides are from 45 different proteins. The intracellular localisation of these proteins was obtained from the UniProt database (www.uniprot.org/) and revealed that of the 9 proteins showing increased oxidation of all detected cysteine residues, 5 (54%) are located to mitochondria and 4 to other parts of the muscle fiber, while of the 20 proteins showing increased reduction of all cysteine residues detected, 19 (95%) are located to the cytosol (95%) and 1 to mitochondria. Thus, these data also support the possibility that redox-sensitive cysteines in proteins in the cytosolic compartment of skeletal muscle fibers from older mice prominently show increased reduction of specific protein cysteines compared with muscles from adult mice, whereas redox sensitive protein cysteines in the mitochondria tend to show greater oxidation.

## 12 Implications for adaptations and aging responses in muscle: potential mechanisms by which H_2_O_2_ might act to stimulate signalling pathways at the low intracellular concentrations found in contracting muscle fibers *in vivo*

There are various potential ways by which physiological (nM) levels of H_2_O_2_ might oxidise cysteine thiols in redox-sensitive proteins [105]. The first is by direct oxidation and some authors have argued that this is facilitated by proximity of the target protein to the source of generation of H_2_O_2_ where local concentrations may be increased [106] (illustrated in Fig. 1), although computational modelling has indicated that even near sites of H_2_O_2_ generation the concentrations are far too low to oxidise typical redox targets [107]. The “floodgate” hypothesis proposes that local scavengers of H_2_O_2_ become rapidly oxidised and inactivated subsequently permitting a local increase in local H_2_O_2_ concentration to micromolar levels.

**Fig. 5:**
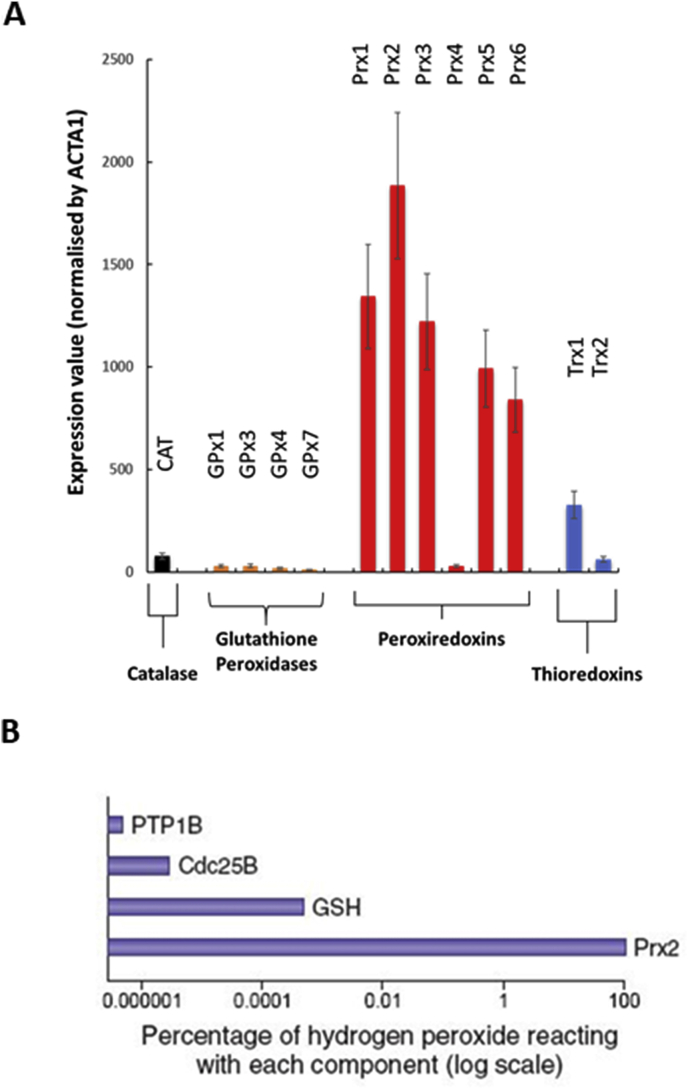
**A**. Relative abundance of key H_2_O_2_ -metabolizing enzymes in single type II skeletal muscle fibers. Redrawn from data published by Murgia et al. [108]. **B**. Selectivity of hydrogen peroxide for peroxiredoxins, protein tyrosine phosphatases and GSH at estimated cellular concentrations, reproduced from Winterbourn [110]. Calculations were performed using competition kinetic analysis. The ratio (*r*) of the amounts reacting with two substrates is derived from the second-order rate equation and is given by the expression *r* = *k*_1_ [substrate 1]/*k*_2_ [substrate 2]. With *n* substrates, the proportion of an oxidant reacting with substrate 1 is given by *k*_1_ [substrate 1]/Σ*k*_n_ [substrate *n*]. Concentrations and rate constants: GSH, 2 mM and 0.89 M^−1^ s^−1^; human peroxiredoxin 2 (Prx2), 20 μM and 2 × 10^7^ M^−1^ s^−1^; protein tyrosine phosphatase 1B (PTP1B), 0.1 μM and 20 M^−1^ s^−1^; cell cycle–activating phosphatase Cdc25B, 0.1 μM and 160 M^−1^ s^−1^.

The second possibility involves the utilisation of highly oxidizable effectors of redox signalling in a “redox relay” that allows transmission of the oxidising equivalents from H_2_O_2_ to less oxidizable target signalling molecules. There are relatively few proteins that are capable of undertaking reaction with H_2_O_2_ with the required sensitivity and a comparison of the abundance of potential candidate proteins (Prx, Trx, gluathione peroxidases [GPx] and catalase) in skeletal muscle fibers is presented in Fig. 5. This mass spectrometry data was obtained from analysis of single type II muscle fibers by Murgia et al. [108] and illustrates the dramatic differences in muscle content of these proteins and the preponderance of Prx enzymes. Prx are a family of enzymes which reduce hydroperoxides to water and are classified by the number of cysteine (Cys) residues involved in the peroxidase activity. The 2-Cys Prxs form a disulphide bond by reacting with peroxides. When acting as a scavenger of H_2_O_2,_ this disulphide has been assumed to be reduced by Trx which is then reduced by Trx reductase (TrxR) [109]. In contrast to the relatively poor reactivity of the key cysteines in proteins in the typical signalling pathways such as MAPK, PTP, PPAR-γ or NF-кB, Prx are several orders of magnitude more reactive with H_2_O_2_ [35] and act to scavenge H_2_O_2_ at the low concentrations found in contracting muscle fibers. Winterbourn has undertaken calculations of the selectivity of hydrogen peroxide for peroxiredoxins in comparison with redox-sensitive signalling proteins such as PTPs and GSH at estimated cellular concentrations and estimated that Prx reacts with almost all of the peroxide (Fig. 5B) [110], further concluding that “the oxidation (of PTPs and related enzymes) observed in cells is likely to be an indirect effect of peroxide reacting with a primary sensor”.

Some studies have also recently indicated that Prxs can function as a signal peroxidase to activate specific pathways. Prx1 activates the transcription factor ASK1 (apoptosis signal-regulating kinase 1) [111] and Prx2 forms a “redox relay” with the transcription factor STAT3 such that oxidative equivalents flow from Prx2 to STAT3 (signal transducer and activator of transcription 3) by generating disulphide-linked STAT3 oligomers with modified transcriptional activity [35].

## 13 Peroxiredoxins are transiently oxidised during contractile activity in skeletal muscle

**Fig. 6:**
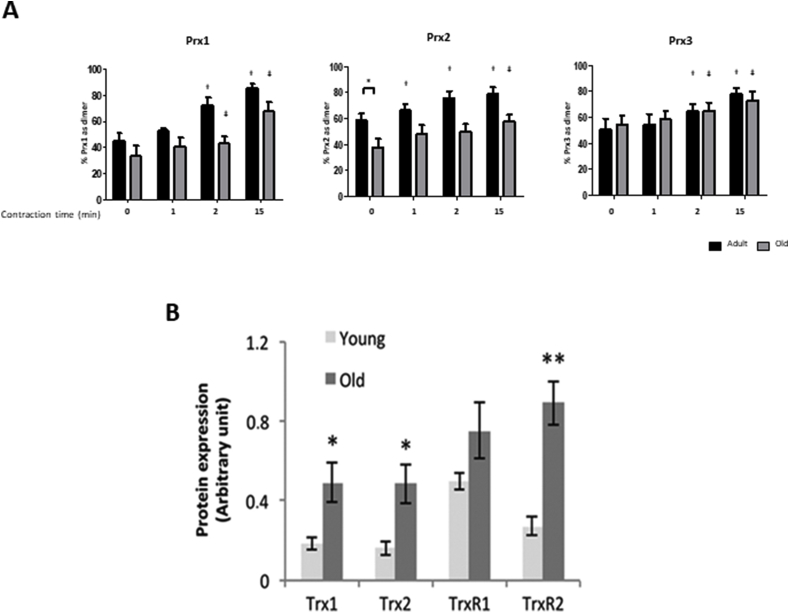
**A**. Proportion of Prx1, 2 and 3 in the oxidised (dimerised) form on denaturing gels from isolated fibers from the FDB of adult and old WT mice subjected to an electrical stimulation protocol for up to 15 min. Fibers were processed at the stated time points by incubation in 75 mM MMTS for 10 min and then lysed in the presence of MMTS. Lysates were subjected to Western blot analysis using non-reducing gels and the Prx antibodies. Results are presented as the percentage of the total Prx signal found in the dimerised form. Graphs show mean ± SEM. *P < 0.05, †P < 0.05 compared to adult baseline values, ƚ P < 0.05 compared to old baseline values. **B**. Published data from our group [63] showing the increase in Trx and TrxR in muscle from old compared with adult mice, *P < 0.05, **P < 0.001 compared with adult.

In recent studies we have examined whether Prx oxidation might mediate responses of muscle to contraction-induced H_2_O_2_. The effects were examined in an isolated FDB fiber model. Following commencement of contractile activity in fibers from adult mice, Prx1, 2 and 3 oxidised rapidly (shown by formation of dimers on non-reducing western blots) (Fig. 6A, [102]). The contraction protocol used had been previously shown to induce adaptions including increased expression of cytoprotective and “antioxidant” proteins in skeletal muscle. For Prx2, this oxidation occurred within 1 min of contractions (Fig. 6A) and the extent of oxidation was significant for all 3 isoforms by the end of the 15 min protocol. Furthermore, the increased Prx oxidation was rapidly reversed following cessation of contractions. No contraction-induced formation of hyperoxidised Prx was seen. In contrast to these studies showing rapid oxidation of Prx in contracting skeletal muscle, our previous work found no evidence for a similar effect on Trx oxidation [63] and taken together these data are compatible with the rapid and specific oxidation of Prx isoforms by physiological concentrations of H_2_O_2_ generated in skeletal muscle during contractile activity.

## 14 Effect of aging on prx oxidation in contracting skeletal muscle fibers

The effect of contractile activity on Prx oxidation was also examined in FDB fibers from old mice in comparison with the data obtained from FDB fibers of adult mice (Fig. 6A). As previously mentioned, the baseline level of oxidation of Prx2 was significantly lower in muscle fibers from old compared with those from adult mice. Oxidation of the protein during contractile activity was also much slower in muscles from old compared with adult mice, such that the proportion of Prx2 in the oxidised form remained lower than the adult baseline mean value at all time points. In fibers from old mice the Prx2 oxidation showed a significant increase above baseline values at 15 min post-contractions compared with 1 min in fibers from adult mice. Prx1 and Prx3 oxidation in fibers from old mice showed the same pattern of response to contractions as seen in fibers from adult mice (Fig. 6A [102]).

This decrease in contraction-induced Prx2 oxidation in muscle fibers from old mice is consistent with the partial disruption of cell signalling and transcription factor activation [10] and supports the hypothesis that Prx activation may be an early step in exercise-induced signalling. As previously discussed, the degree of oxidation of cytosolic Prx2 in quiescent fibers was significantly decreased in muscle from old mice compared with those from adult mice at rest. Baseline levels of the other cytosolic Prx isoform (Prx1) showed a tendency to be reduced and mitochondrial Prx3 remained unchanged in fibers from old compared with adult mice. We speculate that Trx1 may play a role in maintaining Prxs 1 and 2 in the reduced form in this situation since we have previously shown that the contents of both the cytosolic and mitochondrial forms of Trx are elevated in muscle tissue from old mice compared with those from younger mice [63] (Fig. 6B).

## 15 Hypothesis

**Fig. 7:**
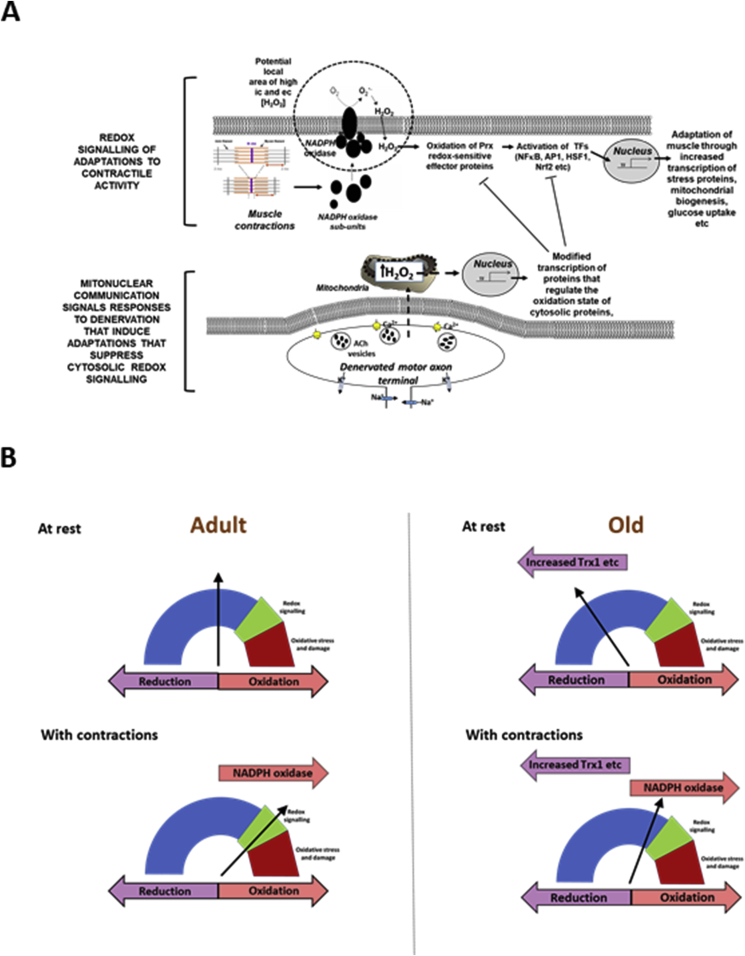
**A.** Schematic representation of how peroxides generated in muscle mitochondria in response to denervation may suppress redox signalling of adaptations to contractions. It was originally envisaged that peroxides generated in mitochondria as a result of denervation might directly act on NADPH oxidase mediated redox signalling pathways, but compartmentalisation prevents such direct interference. It is proposed that the increased mitochondrial stress triggers reverse mitonuclear communication that leads to upregulation of regulatory pathways, such as increased Trx, GPx1, catalase [60] and Prx [63], that causes an increased reductive environment and suppression of redox-sensitive contraction-induced adaptations. **B**. Hypothetical representation of the redox status of critical cysteines involved in signalling responses to contractions in proteins such as the “peroxidatic” cysteine in Prx2. **In adult mice** at rest the redox balance reflects baseline generation of H_2_O_2_ and other oxidising agents and the local amount and activity of reductants and regulatory proteins. During exercise activation of NADPH oxidase leads to a shift to a more oxidised status which is sufficient to lead to oxidation of further signalling proteins potentially through a redox relay system. **In old mice** at rest the redox status of the critical cysteine is shifted to a more reduced state due to the adaptive upregulation of proteins, such as Trx1 which reduce the critical cysteine. During exercise in old mice the oxidation stimulus achieved by activation of NADPH oxidase is therefore insufficient to modify the cysteine sufficiently to activate the signalling pathways.
• The increased peroxide within mitochondria does not lead to an increased cytosolic peroxide, but the increases in adaptive cytoprotective proteins include some located to the muscle cytosol which paradoxically modify the local cytosol redox environment to induce a more reductive state in key cysteines of specific signalling proteins.
• Key adaptations of skeletal muscle to exercise involve transient Prx oxidation as effectors of redox signalling in the cytosol. This requires sensitive oxidation of key cysteine residues. In aging, the chronic change to a more reductive cytosolic environment prevents the appropriate transient contraction-induced oxidation of key cysteines on Prx2 and hence prevents essential adaptations to exercise, thus contributing to loss of muscle mass and function (shown schematically in Fig. 7B).

It is apparent from the data discussed that many conventional ideas about the role of oxidation and increased ROS in aging being mediated solely by increased oxidative damage cannot be sustained. The link between denervation-induced propagation of increased muscle mitochondrial peroxide generation, the apparent compartmentalisation of redox pathways in muscle, and the recognition that Prx are likely facilitators of redox signalling pathways that mediate key adaptations to maintain muscle mass and function lead to an alternative hypothesis of the mechanisms underlying attenuated redox responses to exercise in older individuals:

• Transient loss of integrity of peripheral motor neurons occurs repeatedly throughout life, but is normally rapidly repaired by reinnervation, including by collateral innervation from sprouting axons from neighbouring muscle fibers.
• Each transient loss of neuromuscular integrity leads to a large increase in mitochondrial peroxide production in the denervated fibers and in neighbouring fibers. This peroxide initially acts to stimulate axonal sprouting and regeneration to facilitate rapid reinnervation of the denervated fiber, but also leads to minor oxidative damage and increased expression of a range of cytoprotective proteins as a means of protecting the fiber and neighbouring tissues against severe oxidative damage (Fig. 7A).

**Fig. 8:**
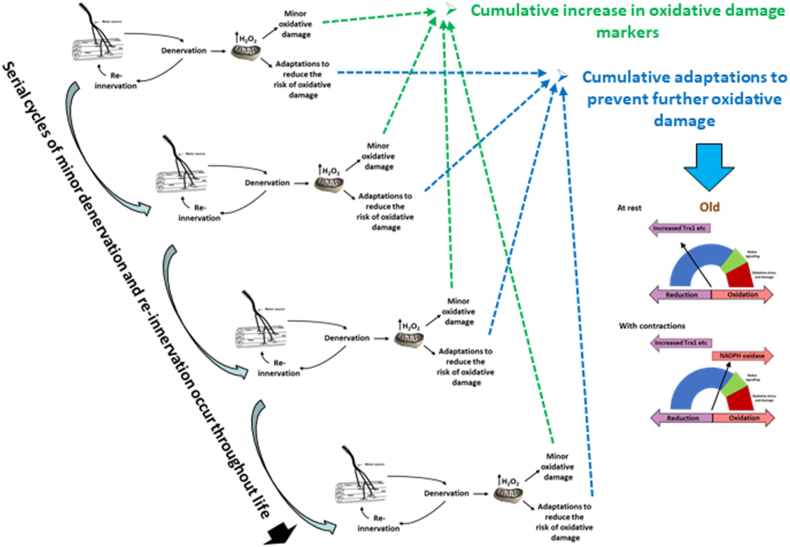
It is envisaged that small episodes of denervation, such as loss of single terminal axons occur frequently throughout life and that this is rapidly repaired by axonal sprouting and outgrowth. Each cycle of denervation and re-innervation leads to a transient increase in mitochondrial peroxide generation in the denervated and neighbouring muscle fibers which cause minor oxidative damage and induction of adaptative responses to prevent further oxidative damage. The repetition of this process many times over a lifetime eventually leads to the cumulative increase in oxidative damage seen in muscle tissue of older animals and man, and also to a cumulative adaptive increase in regulatory proteins designed to prevent oxidative damage, including those in the cytosol that cause a greater reduction in the redox status of critical cysteines (as in Fig. 7B). These changes thus lead to the attenuated redox responses to exercise seen in muscle from old mice and humans which compromises the ability to maintain muscle mass and function in old age.

A schematic diagram to illustrate the interaction of the different aspects of this hypothesis is presented in Fig. 8.

## 16 Testing the hypothesis

The hypothesis described has multiple steps and studies to validate the hypothesis could encompass observational studies in models or situations where muscle loss is reduced in the elderly, or direct interventions to test the hypothesis.

One example situation from which more information may be obtained is study of Masters Athletes in whom muscle function may be well preserved into older age [112]. Such subjects still show an underlying age-related loss of muscle mass and function, but this extent of the loss appears reduced by the regular exercise [113]. There is some evidence that the preservation of muscle in masters athletes is associated with attenuated indices of denervation and greater re-innervation capacity [19] which could reduce the consequences of the repeated episodes of denervation proposed earlier, but examination of the relative rates of mitochondrial peroxide production or of the redox status of the crucial cysteines involved in signalling does not appear to have been undertaken in such subjects.

It was previously mentioned that ageing is associated with apparent preservation of slower twitch (type 1 and 2A) muscle fibers and it could therefore be informative to study whether the hypothesis is sustained in slow twitch vs. fast twitch (type 2B) muscle fibers. The relative preservation of slow fibers in older individuals appears to be associated with less loss of the small motor neurons that innervate slow twitch muscle fibers in comparison with a greater loss of large fast α-motor neurons that innervate fast twitch muscle fibers [19]. Type 1 and 2A slow twitch muscle fibres also contain greater numbers of mitochondria than fast twitch fibres which suggests the mitochondrial peroxide generation may be increased by denervation, but conversely there is evidence that mitochondria in these oxidative fibres generate less H_2_O_2_ per unit mitochondria than those from fast twitch fibres [114]. Thus, these oxidative fibers may be protected from denervation and their mitochondria may have different redox responses to denervation, but this does not appear to have been examined in detail and neither has the redox status of key cysteines involved in signalling.

It is also implied from the hypothesis that interventions at multiple levels should preserve the essential adaptations to exercise that underpins improved maintenance of skeletal muscle mass and function during aging. Many previous studies have examined the possibility that loss of muscle mass and function in aging can be prevented by decreasing muscle oxidation through use of “antioxidants”, but our data indicate that key proteins in the cytosol of muscle are more reduced in old mice and this may attenuate redox-mediated responses to contractile activity. Thus, conventional non-specific antioxidants could not work in this situation.

Some logical interventions in the pathway hypothesised (e.g. prevention of motor neuron loss) are generic and, if feasible, would most likely preserve muscle mass and function during aging whatever the downstream mechanisms. Direct testing of the hypothesis will therefore require more specific interventions:

## 17 Reduction in muscle mitochondrial peroxide generation

One such approach would be organelle-targeted reduction in mitochondrial peroxide production (e.g. through genetic manipulation of mitochondrial catalase, Prx3 or GPx) to prevent the induction of adaptations that lead to increased reduction of cysteines in the cytoplasm. There is also increasing interest in mitochondria-targeted therapeutics that target mitochondrial production of H_2_O_2_ and other peroxides. One such approach is based the mito-targeted peptide SS-31 (also known as *Bendavia* or *Elamipretide* in human therapeutic studies). This peptide rapidly and freely crosses the skeletal muscle plasma membrane and concentrates greater than 1000 fold in the mitochondria. It has been reported to improve muscle mitochondrial bioenergetics and exercise endurance capacity in old mice in addition to reducing muscle mitochondrial H_2_O_2_ generation and increasing the reduced glutathione status [115]. In our previous studies SS-31 administration improved mitochondrial function in old mice but did not affect ageing-associated loss of muscle mass [116]. Other compounds targeted to mitochondria such as mitoquinone mesylate (also known as *MitoQ*) act as mitochondria-selective antioxidants to scavenge ROS within the mitochondrial matrix and provide an alternative route to test the hypothesis [117].

## 18 Reduction in cytosolic Trx1 content

Following this same approach, a potential mechanism can be identified from current data that may lead to the adaptive increased reduction of the cytosol and provides an intervention target to test the hypothesis. We previously reported an elevation in Trx1 (cytosolic) content in muscle from old in comparison with adult mice (Fig. 6B [19]) and the increase in Trx1 provides a potential mechanism leading to greater reduction of Prx1, 2 and other cytosolic redox sensitive proteins that supresses their oxidation following NADPH oxidase activation (Fig. 6). Potential approaches could involve studies of the effect of transgenic knock down of Trx1 or TrxR1 and/or inhibitors of TrxR1 (such as Auranofin) or Trx1 (using PX-12) [118].

## References

[1] Leveille S.G. (2004). Musculoskeletal aging. Curr. Opin. Rheumatol..

[2] Lee I.M., Shiroma E.J., Lobelo F., Puska P., Blair S.N., Katzmarzyk P.T. (2012). Effect of physical inactivity on major non-communicable diseases worldwide: an analysis of burden of disease and life expectancy. Lancet.

[3] Laurin D., Verreault R., Lindsay J., MacPherson K., Rockwood K. (2001). Physical activity and risk of cognitive impairment and dementia in elderly persons. Arch. Neurol..

[4] Young A., Skelton D.A. (1994). Applied physiology of strength and power in old age. Int. J. Sports Med..

[5] Porter M.M., Vandervoort A.A., Lexell J. (1995). Aging of human muscle: structure, function and adaptability. Scand. J. Med. Sci. Sports.

[6] Brooks S.V., Faulkner J.A. (1988). Contractile properties of skeletal muscles from young, adult and aged mice. J. Physiol..

[7] Lexell J., Downham D., Sjöström M. (1986). Distribution of different fibre types in human skeletal muscles. Fibre type arrangement in m. vastus lateralis from three groups of healthy men between 15 and 83 years. J. Neurol. Sci..

[8] Lexell J., Taylor C.C., Sjöström M. (1988). What is the cause of the ageing atrophy? Total number, size and proportion of different fiber types studied in whole vastus lateralis muscle from 15- to 83-year-old men. J. Neurol. Sci..

[9] Demontis F., Piccirillo R., Goldberg A.L., Perrimon N. (2013). Mechanisms of skeletal muscle aging: insights from Drosophila and mammalian models. Dis Model Mech.

[10] Cobley J.N., Sakellariou G.K., Owens D.J., Murray S., Waldron S., Gregson W., Fraser W.D., Burniston J.G., Iwanejko L.A., McArdle A., Morton J.P., Jackson M.J., Close G.L. (2014). Lifelong training preserves some redox-regulated adaptive responses after an acute exercise stimulus in aged human skeletal muscle. Free Radic. Biol. Med..

[11] Campbell M.J., McComas A.J., Petito F. (1973). Physiological changes in ageing muscles. J. Neurol. Neurosurg. Psychiatry.

[12] Sheth K.A., Iyer C.C., Wier C.G., Crum A.E., Bratasz A., Kolb S.J., Clark B.C., Burghes A.H.M., Arnold W.D. (2018). Muscle strength and size are associated with motor unit connectivity in aged mice. Neurobiol. Aging.

[13] Delbono O. (2003). Neural control of aging skeletal muscle. Aging Cell.

[14] Larsson L., Ansved T. (1995). Effects of ageing on the motor unit. Prog. Neurobiol..

[15] Larsson L. (1995). Motor units: remodeling in aged animals. J Gerontol A Biol Sci Med Sci 50 Spec No.

[16] Matthews G.D., Huang C.L., Sun L., Zaidi M. (2011). Translational musculoskeletal science: is sarcopenia the next clinical target after osteoporosis?. Ann. N. Y. Acad. Sci..

[17] Rowan S.L., Rygiel K., Purves-Smith F.M., Solbak N.M., Turnbull D.M., Hepple R.T. (2012). Denervation causes fiber atrophy and myosin heavy chain co-expression in senescent skeletal muscle. PloS One.

[18] Tomlinson B.E., Irving D. (1977). The numbers of limb motor neurons in the human lumbosacral cord throughout life. J. Neurol. Sci..

[19] Sonjak V., Jacob K., Morais J.A., Rivera-Zengotita M., Spendiff S., Spake C., Taivassalo T., Chevalier S., Hepple R.T. (2019). Fidelity of muscle fibre reinnervation modulates ageing muscle impact in elderly women. J. Physiol..

[20] Narici M.V., Maffulli N. (2010). Sarcopenia: characteristics, mechanisms and functional significance. Br. Med. Bull..

[21] Vasilaki A., Pollock N., Giakoumaki I., Goljanek-Whysall K., Sakellariou G.K., Pearson T., Kayani A., Jackson M.J., McArdle A. (2016). The effect of lengthening contractions on neuromuscular junction structure in adult and old mice. Age.

[22] Ham D.J., Börsch A., Lin S., Thürkauf M., Weihrauch M., Reinhard J.R., Delezie J., Battilana F., Wang X., Kaiser M.S., Guridi M., Sinnreich M., Rich M.M., Mittal N., Tintignac L.A., Handschin C., Zavolan M., Rüegg M.A. (2020). The neuromuscular junction is a focal point of mTORC1 signaling in sarcopenia. Nat. Commun..

[23] Marzuca-Nassr G.N., SanMartín-Calísto Y., Guerra-Vega P., Artigas-Arias M., Alegría A., Curi R. (2020). Skeletal muscle aging atrophy: assessment and exercise-based treatment. Adv. Exp. Med. Biol..

[24] de Mello R.G.B., Dalla Corte R.R., Gioscia J., Moriguchi E.H. (2019). Effects of physical exercise programs on sarcopenia management, dynapenia, and physical performance in the elderly: a systematic review of randomized clinical trials. J Aging Res.

[25] Gabriel B.M., Zierath J.R. (2017). The limits of exercise physiology: from performance to health. Cell Metabol..

[26] Powers S.K., Jackson M.J. (2008). Exercise-induced oxidative stress: cellular mechanisms and impact on muscle force production. Physiol. Rev..

[27] Jackson M.J., Vasilaki A., McArdle A. (2016). Cellular mechanisms underlying oxidative stress in human exercise. Free Radic. Biol. Med..

[28] Jackson M.J. (2005). Reactive oxygen species and redox-regulation of skeletal muscle adaptations to exercise. Philos. Trans. R. Soc. Lond. B Biol. Sci..

[29] Palomero J., Pye D., Kabayo T., Spiller D.G., Jackson M.J. (2008). In situ detection and measurement of intracellular reactive oxygen species in single isolated mature skeletal muscle fibers by real time fluorescence microscopy. Antioxidants Redox Signal..

[30] Pye D., Palomero J., Kabayo T., Jackson M.J. (2007). Real-time measurement of nitric oxide in single mature mouse skeletal muscle fibres during contractions. J. Physiol..

[31] Droge W. (2002). Free radicals in the physiological control of cell function. Physiol. Rev..

[32] Haddad J.J. (2002). Oxygen-sensing mechanisms and the regulation of redox-responsive transcription factors in development and pathophysiology. Respir. Res..

[33] Jackson M.J., Papa S., Bolanos J., Bruckdorfer R., Carlsen H., Elliott R.M., Flier J., Griffiths H.R., Heales S., Holst B., Lorusso M., Lund E., Oivind Moskaug J., Moser U., Di Paola M., Polidori M.C., Signorile A., Stahl W., Vina-Ribes J., Astley S.B. (2002). Antioxidants, reactive oxygen and nitrogen species, gene induction and mitochondrial function. Mol. Aspect. Med..

[34] Janssen-Heininger Y.M., Mossman B.T., Heintz N.H., Forman H.J., Kalyanaraman B., Finkel T., Stamler J.S., Rhee S.G., van der Vliet A. (2008). Redox-based regulation of signal transduction: principles, pitfalls, and promises. Free Radic. Biol. Med..

[35] Sobotta M.C., Liou W., Stocker S., Talwar D., Oehler M., Ruppert T., Scharf A.N., Dick T.P. (2015). Peroxiredoxin-2 and STAT3 form a redox relay for H2O2 signaling. Nat. Chem. Biol..

[36] Khassaf M., McArdle A., Esanu C., Vasilaki A., McArdle F., Griffiths R.D., Brodie D.A., Jackson M.J. (2003). Effect of vitamin C supplements on antioxidant defence and stress proteins in human lymphocytes and skeletal muscle. J. Physiol..

[37] Venditti P., Napolitano G., Barone D., Di Meo S. (2014). Vitamin E supplementation modifies adaptive responses to training in rat skeletal muscle. Free Radic. Res..

[38] Ristow M., Zarse K., Oberbach A., Kloting N., Birringer M., Kiehntopf M., Stumvoll M., Kahn C.R., Bluher M. (2009). Antioxidants prevent health-promoting effects of physical exercise in humans. Proc. Natl. Acad. Sci. U. S. A..

[39] Paulsen G., Cumming K.T., Holden G., Hallen J., Ronnestad B.R., Sveen O., Skaug A., Paur I., Bastani N.E., Ostgaard H.N., Buer C., Midttun M., Freuchen F., Wiig H., Ulseth E.T., Garthe I., Blomhoff R., Benestad H.B., Raastad T. (2014). Vitamin C and E supplementation hampers cellular adaptation to endurance training in humans: a double-blind, randomised, controlled trial. J. Physiol..

[40] Gomez-Cabrera M.C., Domenech E., Romagnoli M., Arduini A., Borras C., Pallardo F.V., Sastre J., Vina J. (2008). Oral administration of vitamin C decreases muscle mitochondrial biogenesis and hampers training-induced adaptations in endurance performance. Am. J. Clin. Nutr..

[41] Wuyts W.A., Vanaudenaerde B.M., Dupont L.J., Demedts M.G., Verleden G.M. (2003). N-acetylcysteine reduces chemokine release via inhibition of p38 MAPK in human airway smooth muscle cells. Eur. Respir. J..

[42] Gomez-Cabrera M.C., Ristow M., Vina J. (2012). Antioxidant supplements in exercise: worse than useless?. Am. J. Physiol. Endocrinol. Metab..

[43] Higashida K., Kim S.H., Higuchi M., Holloszy J.O., Han D.H. (2011). Normal adaptations to exercise despite protection against oxidative stress. Am. J. Physiol. Endocrinol. Metab..

[44] Jackson M.J., Stretton C., McArdle A. (2020). Hydrogen peroxide as a signal for skeletal muscle adaptations to exercise: what do concentrations tell us about potential mechanisms?. Redox Biol.

[45] Gloire G., Piette J. (2009). Redox regulation of nuclear post-translational modifications during NF-kappaB activation. Antioxidants Redox Signal..

[46] Zhang J., Johnston G., Stebler B., Keller E.T. (2001). Hydrogen peroxide activates NFkappaB and the interleukin-6 promoter through NFkappaB-inducing kinase. Antioxidants Redox Signal..

[47] Bassi R., Burgoyne J.R., DeNicola G.F., Rudyk O., DeSantis V., Charles R.L., Eaton P., Marber M.S. (2017). Redox-dependent dimerization of p38alpha mitogen-activated protein kinase with mitogen-activated protein kinase kinase 3. J. Biol. Chem..

[48] Marinho H.S., Real C., Cyrne L., Soares H., Antunes F. (2014). Hydrogen peroxide sensing, signaling and regulation of transcription factors. Redox biology.

[49] Sies H. (2014). Role of metabolic H2O2 generation: redox signaling and oxidative stress. J. Biol. Chem..

[50] Jackson M.J., McArdle A. (2011). Age-related changes in skeletal muscle reactive oxygen species generation and adaptive responses to reactive oxygen species. J. Physiol..

[51] Pomatto L.C.D., Davies K.J.A. (2017). The role of declining adaptive homeostasis in ageing. J. Physiol..

[52] Vasilaki A., McArdle F., Iwanejko L.M., McArdle A. (2006). Adaptive responses of mouse skeletal muscle to contractile activity: the effect of age. Mech. Ageing Dev..

[53] Ljubicic V., Hood D.A. (2008). Kinase-specific responsiveness to incremental contractile activity in skeletal muscle with low and high mitochondrial content. Am. J. Physiol. Endocrinol. Metab..

[54] Viña J., Gomez-Cabrera M.C., Borras C., Froio T., Sanchis-Gomar F., Martinez-Bello V.E., Pallardo F.V. (2009). Mitochondrial biogenesis in exercise and in ageing. Adv. Drug Deliv. Rev..

[55] Cuthbertson D., Smith K., Babraj J., Leese G., Waddell T., Atherton P., Wackerhage H., Taylor P.M., Rennie M.J. (2005). Anabolic signaling deficits underlie amino acid resistance of wasting, aging muscle. Faseb. J..

[56] McArdle A., Dillmann W.H., Mestril R., Faulkner J.A., Jackson M.J. (2004). Overexpression of HSP70 in mouse skeletal muscle protects against muscle damage and age-related muscle dysfunction. Faseb. J..

[57] Broome C.S., Kayani A.C., Palomero J., Dillmann W.H., Mestril R., Jackson M.J., McArdle A. (2006). Effect of lifelong overexpression of HSP70 in skeletal muscle on age-related oxidative stress and adaptation after nondamaging contractile activity. Faseb. J..

[58] Kayani A.C., Close G.L., Dillmann W.H., Mestril R., Jackson M.J., McArdle A. (2010). Overexpression of HSP10 in skeletal muscle of transgenic mice prevents the age-related fall in maximum tetanic force generation and muscle Cross-Sectional Area. Am. J. Physiol. Regul. Integr. Comp. Physiol..

[59] Cobley J.N., Moult P.R., Burniston J.G., Morton J.P., Close G.L. (2015). Exercise improves mitochondrial and redox-regulated stress responses in the elderly: better late than never!. Biogerontology.

[60] Palomero J., Vasilaki A., Pye D., McArdle A., Jackson M.J. (2013). Aging increases the oxidation of dichlorohydrofluorescein in single isolated skeletal muscle fibers at rest, but not during contractions. Am. J. Physiol. Regul. Integr. Comp. Physiol..

[61] Vasilaki A., van der Meulen J.H., Larkin L., Harrison D.C., Pearson T., Van Remmen H., Richardson A., Brooks S.V., Jackson M.J., McArdle A. (2010). The age-related failure of adaptive responses to contractile activity in skeletal muscle is mimicked in young mice by deletion of Cu,Zn superoxide dismutase. Aging Cell.

[62] Martinez Guimera A., Welsh C.M., Proctor C.J., McArdle A., Shanley D.P. (2018). Molecular habituation' as a potential mechanism of gradual homeostatic loss with age. Mech. Ageing Dev..

[63] Dimauro I., Pearson T., Caporossi D., Jackson M.J. (2012). In vitro susceptibility of thioredoxins and glutathione to redox modification and aging-related changes in skeletal muscle. Free Radic. Biol. Med..

[64] McDonagh B., Sakellariou G.K., Smith N.T., Brownridge P., Jackson M.J. (2014). Differential cysteine labeling and global label-free proteomics reveals an altered metabolic state in skeletal muscle aging. J. Proteome Res..

[65] Vasilaki A., Mansouri A., Van Remmen H., van der Meulen J.H., Larkin L., Richardson A.G., McArdle A., Faulkner J.A., Jackson M.J. (2006). Free radical generation by skeletal muscle of adult and old mice: effect of contractile activity. Aging Cell.

[66] Drew B., Phaneuf S., Dirks A., Selman C., Gredilla R., Lezza A., Barja G., Leeuwenburgh C. (2003). Effects of aging and caloric restriction on mitochondrial energy production in gastrocnemius muscle and heart. Am. J. Physiol. Regul. Integr. Comp. Physiol..

[67] Sastre J., Pallardó F.V., Viña J. (2003). The role of mitochondrial oxidative stress in aging. Free Radic. Biol. Med..

[68] Orr W.C., Sohal R.S. (1993). Effects of Cu-Zn superoxide dismutase overexpression of life span and resistance to oxidative stress in transgenic Drosophila melanogaster. Arch. Biochem. Biophys..

[69] Orr W.C., Sohal R.S. (1994). Extension of life-span by overexpression of superoxide dismutase and catalase in Drosophila melanogaster. Science.

[70] Melov S., Ravenscroft J., Malik S., Gill M.S., Walker D.W., Clayton P.E., Wallace D.C., Malfroy B., Doctrow S.R., Lithgow G.J. (2000). Extension of life-span with superoxide dismutase/catalase mimetics. Science.

[71] Orr W.C., Sohal R.S. (2003). Does overexpression of Cu,Zn-SOD extend life span in Drosophila melanogaster?. Exp. Gerontol..

[72] Gems D., Doonan R. (2009). Antioxidant defense and aging in C. elegans: is the oxidative damage theory of aging wrong?. Cell Cycle.

[73] Hamilton R.T., Walsh M.E., Van Remmen H. (2012). Mouse models of oxidative stress indicate a role for modulating healthy aging. J. Clin. Exp. Pathol..

[74] Jang Y.C., Van Remmen H. (2009). The mitochondrial theory of aging: insight from transgenic and knockout mouse models. Exp. Gerontol..

[75] Brand M.D., Orr A.L., Perevoshchikova I.V., Quinlan C.L. (2013). The role of mitochondrial function and cellular bioenergetics in ageing and disease. Br. J. Dermatol..

[76] Gomez-Cabrera M.C., Sanchis-Gomar F., Garcia-Valles R., Pareja-Galeano H., Gambini J., Borras C., Viña J. (2012). Mitochondria as sources and targets of damage in cellular aging. Clin. Chem. Lab. Med..

[77] Larkin L.M., Davis C.S., Sims-Robinson C., Kostrominova T.Y., Van Remmen H., Richardson A., Feldman E.L., Brooks S.V. (2011). Skeletal muscle weakness due to deficiency of CuZn-superoxide dismutase is associated with loss of functional innervation. Am. J. Physiol. Regul. Integr. Comp. Physiol..

[78] Muller F.L., Song W., Jang Y.C., Liu Y., Sabia M., Richardson A., Van Remmen H. (2007). Denervation-induced skeletal muscle atrophy is associated with increased mitochondrial ROS production. Am. J. Physiol. Regul. Integr. Comp. Physiol..

[79] Pollock N., Staunton C.A., Vasilaki A., McArdle A., Jackson M.J. (2017). Denervated muscle fibers induce mitochondrial peroxide generation in neighboring innervated fibers: role in muscle aging. Free Radic. Biol. Med..

[80] Muller F.L., Song W., Liu Y., Chaudhuri A., Pieke-Dahl S., Strong R., Huang T.T., Epstein C.J., Roberts L.J., Csete M., Faulkner J.A., Van Remmen H. (2006). Absence of CuZn superoxide dismutase leads to elevated oxidative stress and acceleration of age-dependent skeletal muscle atrophy. Free Radic. Biol. Med..

[81] Zhang Y., Davis C., Sakellariou G.K., Shi Y., Kayani A.C., Pulliam D., Bhattacharya A., Richardson A., Jackson M.J., McArdle A., Brooks S.V., Van Remmen H. (2013). CuZnSOD gene deletion targeted to skeletal muscle leads to loss of contractile force but does not cause muscle atrophy in adult mice. Faseb. J..

[82] Sakellariou G.K., Davis C.S., Shi Y., Ivannikov M.V., Zhang Y., Vasilaki A., Macleod G.T., Richardson A., Van Remmen H., Jackson M.J., McArdle A., Brooks S.V. (2014). Neuron-specific expression of CuZnSOD prevents the loss of muscle mass and function that occurs in homozygous CuZnSOD-knockout mice. Faseb. J..

[83] Deepa S.S., Van Remmen H., Brooks S.V., Faulkner J.A., Larkin L., McArdle A., Jackson M.J., Vasilaki A., Richardson A. (2019). Accelerated sarcopenia in Cu/Zn superoxide dismutase knockout mice. Free Radic. Biol. Med..

[84] Sakellariou G.K., McDonagh B., Porter H., Giakoumaki, Earl K.E., Nye G.A., Vasilaki A., Brooks S.V., Richardson A., Van Remmen H., McArdle A., Jackson M.J. (2018). Comparison of whole body SOD1 knockout with muscle-specific SOD1 knockout mice reveals a role for nerve redox signaling in regulation of degenerative pathways in skeletal muscle. Antioxidants Redox Signal..

[85] Bhattacharya A., Muller F.L., Liu Y., Sabia M., Liang H., Song W., Jang Y.C., Ran Q., Van Remmen H. (2009). Denervation induces cytosolic phospholipase A2-mediated fatty acid hydroperoxide generation by muscle mitochondria. J. Biol. Chem..

[86] Bhattacharya A., Hamilton R., Jernigan A., Zhang Y., Sabia M., Rahman M.M., Li Y., Wei R., Chaudhuri A., Van Remmen H. (2014). Genetic ablation of 12/15-lipoxygenase but not 5-lipoxygenase protects against denervation-induced muscle atrophy. Free Radic. Biol. Med..

[87] Rieger S., Sagasti A. (2011). Hydrogen peroxide promotes injury-induced peripheral sensory axon regeneration in the zebrafish skin. PLoS Biol..

[88] Min J.Y., Park M.H., Park M.K., Park K.W., Lee N.W., Kim T., Kim H.J., Lee D.H. (2006). Staurosporin induces neurite outgrowth through ROS generation in HN33 hippocampal cell lines. J. Neural. Transm..

[89] Sidlauskaite E., Gibson J.W., Megson I.L., Whitfield P.D., Tovmasyan A., Batinic-Haberle I., Murphy M.P., Moult P.R., Cobley J.N. (2018). Mitochondrial ROS cause motor deficits induced by synaptic inactivity: implications for synapse pruning. Redox Biol.

[90] Scalabrin M., Pollock N., Staunton C.A., Brooks S.V., McArdle A., Jackson M.J., Vasilaki A. (2019). Redox responses in skeletal muscle following denervation. Redox Biol.

[91] Halliwell B., Gutteridge J.M. (1984). Free radicals, lipid peroxidation, and cell damage. Lancet.

[92] Sakellariou G.K., Vasilaki A., Palomero J., Kayani A., Zibrik L., McArdle A., Jackson M.J. (2013). Studies of mitochondrial and nonmitochondrial sources implicate nicotinamide adenine dinucleotide phosphate oxidase(s) in the increased skeletal muscle superoxide generation that occurs during contractile activity. Antioxidants Redox Signal..

[93] Pearson T., Kabayo T., Ng R., Chamberlain J., McArdle A., Jackson M.J. (2014). Skeletal muscle contractions induce acute changes in cytosolic superoxide, but slower responses in mitochondrial superoxide and cellular hydrogen peroxide. PloS One.

[94] Staunton C.A., Owen E.D., Pollock N., Vasilaki A., Barrett-Jolley R., McArdle A., Jackson M.J. (2019). HyPer2 imaging reveals temporal and heterogeneous hydrogen peroxide changes in denervated and aged skeletal muscle fibers in vivo. Sci. Rep..

[95] Dey S., Sidor A., O'Rourke B. (2016). Compartment-specific control of reactive oxygen species scavenging by antioxidant pathway enzymes. J. Biol. Chem..

[96] Booty L.M., Gawel J.M., Cvetko F., Caldwell S.T., Hall A.R., Mulvey J.F., James A.M., Hinchy E.C., Prime T.A., Arndt S., Beninca C., Bright T.P., Clatworthy M.R., Ferdinand J.R., Prag H.A., Logan A., Prudent J., Krieg T., Hartley R.C., Murphy M.P. (2019). Selective disruption of mitochondrial thiol redox state in cells and in vivo. Cell Chem Biol.

[97] Mottis A., Herzig S., Auwerx J. (2019). Mitocellular communication: shaping health and disease. Science.

[98] Merry T.L., Ristow M. (2016). Mitohormesis in exercise training. Free Radic. Biol. Med..

[99] Pak V.V., Ezerina D., Lyublinskaya O.G., Pedre B., Tyurin-Kuzmin P.A., Mishina N.M., Thauvin M., Young D., Wahni K., Martinez Gache S.A., Demidovich A.D., Ermakova Y.G., Maslova Y.D., Shokhina A.G., Eroglu E., Bilan D.S., Bogeski I., Michel T., Vriz S., Messens J., Belousov V.V. (2020). Ultrasensitive genetically encoded indicator for hydrogen peroxide identifies roles for the oxidant in cell migration and mitochondrial function. Cell Metabol..

[100] Tomalin L.E., Day A.M., Underwood Z.E., Smith G.R., Dalle Pezze P., Rallis C., Patel W., Dickinson B.C., Bähler J., Brewer T.F., Chang C.J., Shanley D.P., Veal E.A. (2016). Increasing extracellular H2O2 produces a bi-phasic response in intracellular H2O2, with peroxiredoxin hyperoxidation only triggered once the cellular H2O2-buffering capacity is overwhelmed. Free Radic. Biol. Med..

[101] Jackson M.J., McArdle A. (2016). Role of reactive oxygen species in age-related neuromuscular deficits. J. Physiol..

[102] Stretton C., Pugh J.N., McDonagh B., McArdle A., Close G.L., Jackson M.J. (2020). 2-Cys peroxiredoxin oxidation in response to hydrogen peroxide and contractile activity in skeletal muscle: a novel insight into exercise-induced redox signalling?. Free Radic. Biol. Med..

[103] Smith N.T., Soriano-Arroquia A., Goljanek-Whysall K., Jackson M.J., McDonagh B. (2018). Redox responses are preserved across muscle fibres with differential susceptibility to aging. J Proteomics.

[104] Held J.M. (2020). Redox systems biology: harnessing the sentinels of the cysteine redoxome. Antioxidants Redox Signal..

[105] Stocker S., Maurer M., Ruppert T., Dick T.P. (2018). A role for 2-Cys peroxiredoxins in facilitating cytosolic protein thiol oxidation. Nat. Chem. Biol..

[106] Ushio-Fukai M. (2009). Compartmentalization of redox signaling through NADPH oxidase-derived ROS. Antioxidants Redox Signal..

[107] Travasso R.D.M., Sampaio Dos Aidos F., Bayani A., Abranches P., Salvador A. (2017). Localized redox relays as a privileged mode of cytoplasmic hydrogen peroxide signaling. Redox biology.

[108] Murgia M., Toniolo L., Nagaraj N., Ciciliot S., Vindigni V., Schiaffino S., Reggiani C., Mann M. (2017). Single muscle fiber proteomics reveals fiber-type-specific features of human muscle aging. Cell Rep..

[109] Park J., Lee S., Lee S., Kang S.W. (2014). 2-cys peroxiredoxins: emerging hubs determining redox dependency of mammalian signaling networks. International journal of cell biology.

[110] Winterbourn C.C. (2008). Reconciling the chemistry and biology of reactive oxygen species. Nat. Chem. Biol..

[111] Jarvis R.M., Hughes S.M., Ledgerwood E.C. (2012). Peroxiredoxin 1 functions as a signal peroxidase to receive, transduce, and transmit peroxide signals in mammalian cells. Free Radic. Biol. Med..

[112] Gries K.J., Minchev K., Raue U., Grosicki G.J., Begue G., Finch W.H., Graham B., Trappe T.A., Trappe S. (1985). Single-muscle fiber contractile properties in lifelong aerobic exercising women. J. Appl. Physiol..

[113] Lazarus N.R., Harridge S.D.R. (2017). Declining performance of master athletes: silhouettes of the trajectory of healthy human ageing?. J. Physiol..

[114] Anderson E.J., Neufer P.D. (2006). Type II skeletal myofibers possess unique properties that potentiate mitochondrial H(2)O(2) generation. Am. J. Physiol. Cell Physiol..

[115] Siegel M.P., Kruse S.E., Percival J.M., Goh J., White C.C., Hopkins H.C., Kavanagh T.J., Szeto H.H., Rabinovitch P.S., Marcinek D.J. (2013). Mitochondrial-targeted peptide rapidly improves mitochondrial energetics and skeletal muscle performance in aged mice. Aging Cell.

[116] Sakellariou G.K., Pearson T., Lightfoot A.P., Nye G.A., Wells N., Giakoumaki, Vasilaki A., Griffiths R.D., Jackson M.J., McArdle A. (2016). Mitochondrial ROS regulate oxidative damage and mitophagy but not age-related muscle fiber atrophy. Sci. Rep..

[117] Sakellariou G.K., Pearson T., Lightfoot A.P., Nye G.A., Wells N., Giakoumaki, Griffiths R.D., McArdle A., Jackson M.J. (2016). Long-term administration of the mitochondria-targeted antioxidant mitoquinone mesylate fails to attenuate age-related oxidative damage or rescue the loss of muscle mass and function associated with aging of skeletal muscle. Faseb. J..

[118] Raninga P.V., Di Trapani G., Vuckovic S., Bhatia M., Tonissen K.F. (2015). Inhibition of thioredoxin 1 leads to apoptosis in drug-resistant multiple myeloma. Oncotarget.

